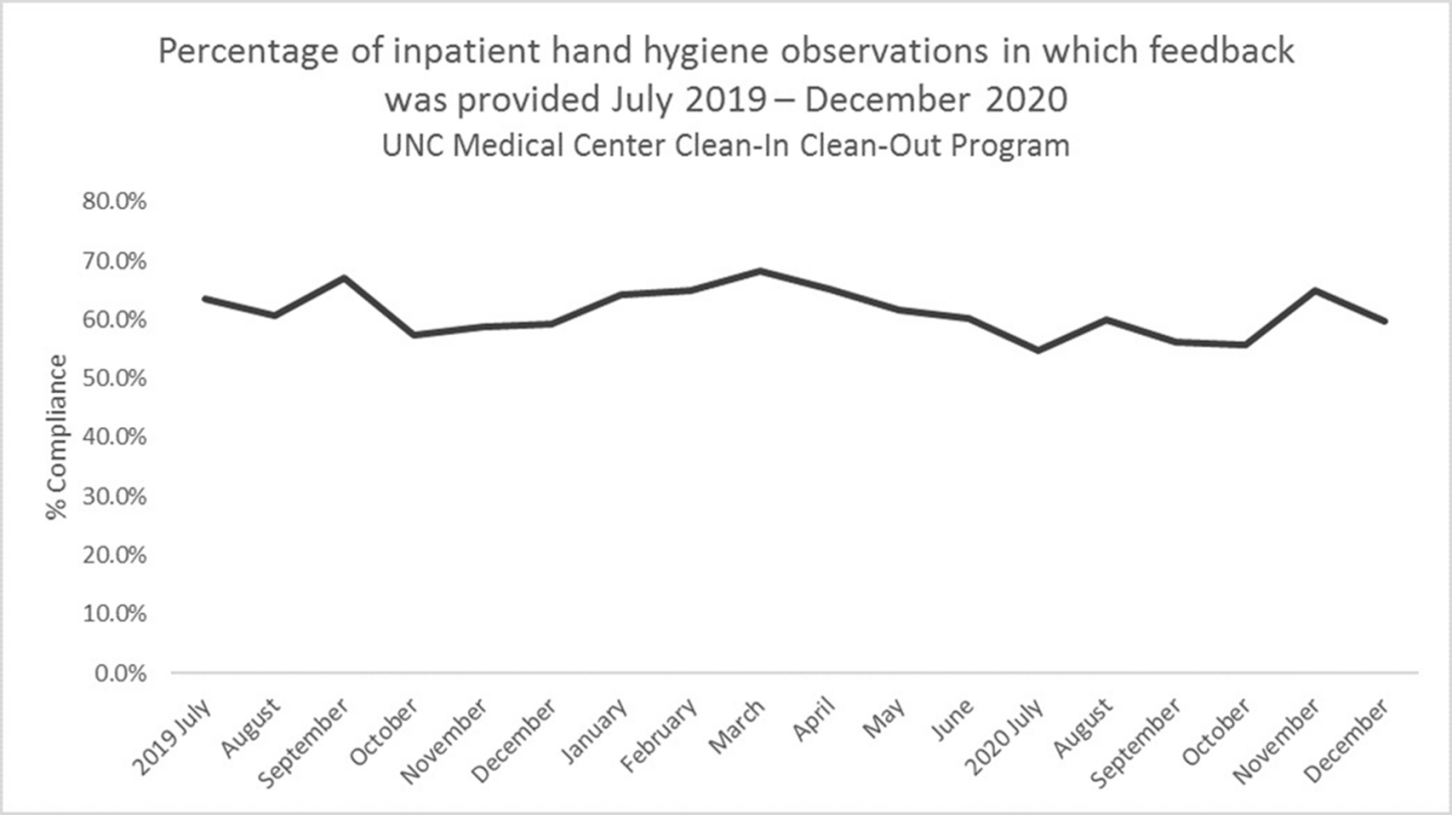# Building on the Foundation of a Sustainable Hand Hygiene Program During the COVID-19 Pandemic

**DOI:** 10.1017/ash.2021.124

**Published:** 2021-07-29

**Authors:** Lisa Stancill, Emily Sickbert-Bennett Vavalle, Lauren DiBiase

## Abstract

**Background:** Hand hygiene is essential to preventing the spread of disease in hospitals. Renewed emphasis has been placed on hand hygiene during the COVID-19 pandemic. We investigated whether UNC Medical Center’s well-established Clean-In Clean-Out (CICO) program for hand hygiene observations was sustainable throughout a public health and healthcare crisis and whether the COVID-19 pandemic had an effect on hand hygiene compliance. **Methods:** UNC Medical Center utilizes a crowd-sourced hand-hygiene audit application, CICO, to track hand-hygiene observations, compliance, and feedback. This application encourages participation from all staff and promotes providing real-time feedback in the form of a compliment or reminder when performing hand hygiene observations. During this evaluation, hand hygiene data were queried from the CICO application on the number of observations performed, hand hygiene compliance percentage, and feedback compliance percentage from July 2019 to December 2020. Hand hygiene data were compared to patient volumes in different care settings and the number of hospitalized patients being treated for COVID-19. **Results:** Initial increases in hand hygiene observations, compliance, and feedback were detected in the months leading up to UNC Medical Center receiving its first SARS-CoV-2–positive patient. Observations were highest when patient volumes were low due to closed clinics and restrictions on elective surgeries (Figure 1). When patient volumes returned to pre–COVID-19 levels coupled with treating more COVID-19 patients, the number of observations and compliance rate metrics declined. Feedback compliance percentage remained relatively stable through the entire period (Figure 2). **Conclusions:** Despite the additional strain on healthcare staff during COVID-19, the CICO model was a sustainable method to track hand hygiene observations and compliance. Notably, however, engagement was highest when patient census was lower, demonstrating that operating at a high capacity is not beneficial for patient safety. Due to the success and sustainment of the CICO program, UNC Medical Center used this model to create a Mask-On Mask-Up campaign to engage staff to submit observations, track compliance, and encourage feedback to promote the appropriate use of masks during COVID-19.

**Funding:** No

**Disclosures:** None

**Figure 1.**
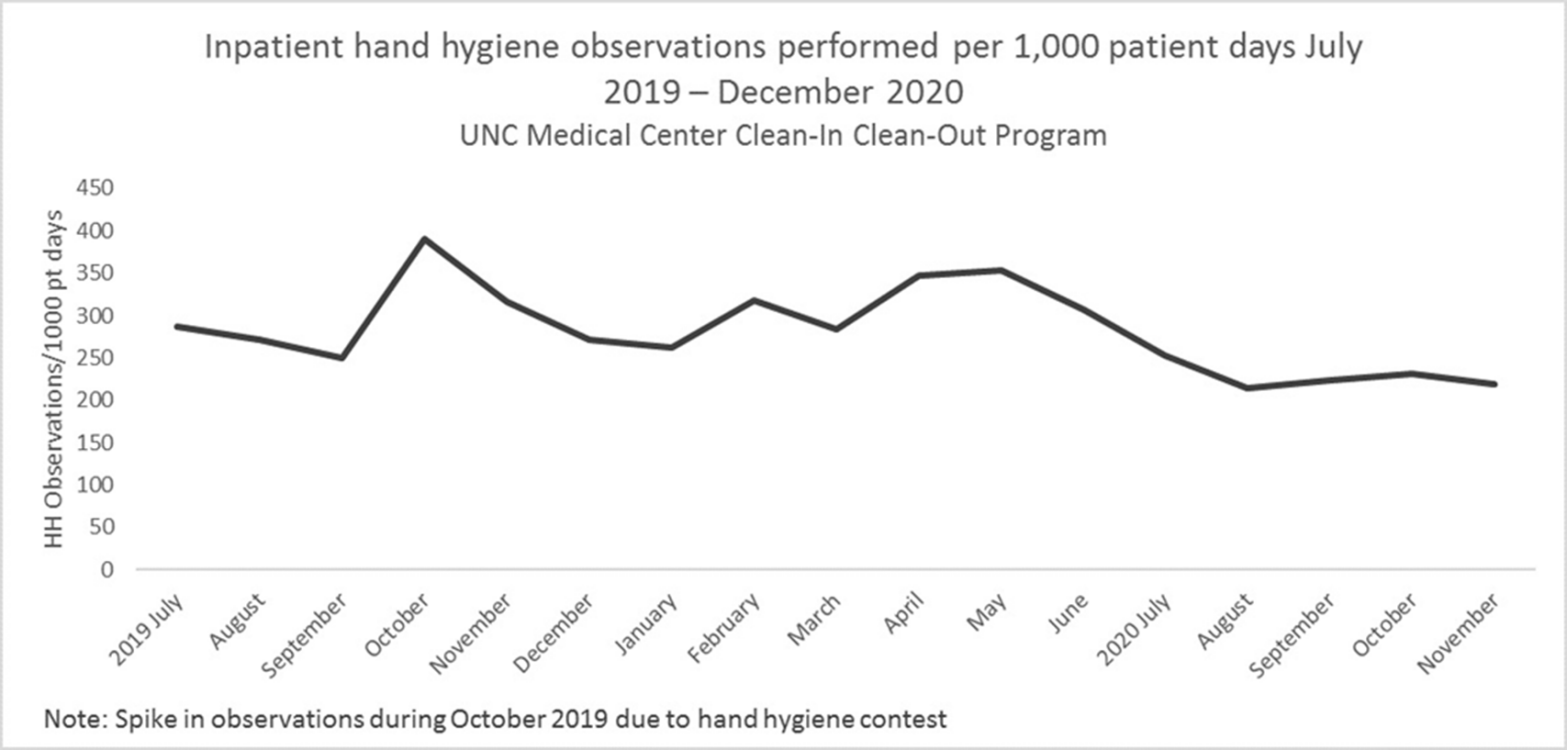

**Figure 2.**